# LINC00152 upregulates ZEB1 expression and enhances epithelial-mesenchymal transition and oxaliplatin resistance in esophageal cancer by interacting with EZH2

**DOI:** 10.1186/s12935-020-01620-1

**Published:** 2020-11-26

**Authors:** Shuyao Zhang, Wei Liao, Qinshui Wu, Xiaoshan Huang, Zhen Pan, Wang Chen, Shuyi Gu, Zuojun Huang, Yiwen Wang, Xu Tang, Shanshan Liang, Xiaoyan Zhang, Yun Chen, Shuang Chen, Wanying Chen, Yi Jiang, Chen Chen, Guodong Qiu

**Affiliations:** 1 Guangzhou Red Cross Hospital Affiliated of Ji-Nan University, Guangzhou, 510220 P.R. China; 2 Department of Pharmacology, Shantou University Medical College, Shantou, 515000 P.R. China; 3 Digestive Oncology, Cancer Hospital of Shantou University Medical College, Shantou, 515000 P.R. China; 4 Department of Pharmacy, Cancer Hospital of Shantou University Medical College, Shantou, 515000 P.R. China

**Keywords:** Esophageal cancer, LINC00152, Zeste Homologue 2, Zinc finger e-box binding homeobox 1, Epithelial-mesenchymal transition, Chemotherapy resistance

## Abstract

**Background:**

Expression of the long non-coding mRNA LINC00152 has been reported to correlate with cancer cell resistance to oxaliplatin (L-OHP). However, little is known regarding the molecular mechanism of LINC00152 in esophageal cancer (EC). Hence, we intended to characterize the role of LINC00152 in EC, with a special focus on epithelial-mesenchymal transition (EMT) and L-OHP resistance.

**Methods:**

We collected EC tissues and identified EC cell lines with higher L-OHP resistance, and then characterized expression patterns of LINC00152, Zeste Homologue 2 (EZH2), Zinc finger e-box binding homeobox (ZEB1) and EMT-related genes using RT-qPCR and Western blot analysis. Furthermore, their functional significance was identified by gain and loss-of-function experiments. The relationship among LINC00152, EZH2 and ZEB1 was examined using RIP, RNA pull-down and ChIP assays. Additionally, resistance of EC cells to L-OHP was reflected by CCK-8 assay to detect cell viability. Animal experiments were also conducted to detect the effects of the LINC00152/EZH2/ZEB1 on EMT and L-OHP resistance.

**Results:**

LINC00152, EZH2 and ZEB1 were highly expressed in EC tissues and Kyse−150/TE-1 cells. As revealed by assays *in vitro* and *in vivo*, LINC00152 positively regulated ZEB1 expression through interaction with EZH2 to enhance EMT and L-OHP resistance in EC cells. In contrast, silencing of LINC00152 contributed to attenuated EMT and drug resistance of EC cells to L-OHP.

**Conclusions:**

Our study demonstrates that LINC00152/EZH2/ZEB1 axis can regulate EMT and resistance of EC cells to L-OHP, thus presenting a potential therapeutic target for EC treatment.

## Background

Esophageal cancer (EC) ranked 6th in incidence and 7th in cancer-related mortality globally in 2018 [1], and is accompanied by high aggressiveness and low survival rate [2]. Esophageal squamous cell carcinoma (ESCC) accounts for approximately 90% of global EC cases each year. [3] Current treatment options to improve survival rates of EC patients include radiotherapy [4], surgery, and chemotherapy [5]. Besides, oxaliplatin (L-OHP) is a promising antineoplastic agent that has been increasingly used in EC [6], although L-OHP resistance presents a major medical problem in this form of EC treatment [7]. Previous work has shown that epithelial-mesenchymal transition (EMT) is associated with chemotherapy resistance [8]. Accumulating studies have reported that long non-coding RNAs (lncRNAs) are key regulators of drug resistance and EMT [9–11].

LncRNAs are a class of RNA transcripts of 200 nucleotides in length with limited protein transcriptional potential [12]. However, lncRNAs, are emerging as key regulators of tumor development and progression, which have been studied intensively in gastric cancer, colon cancer [13] and ESCC [14]. Mounting evidence indicates that lncRNAs could serve as targets for EC treatment based on their roles in influencing resistance of cancer cells to chemotherapy, as is notably described for the lncRNAs PART1 [15], DANCR [16] and LINC01419 [17]. Intriguingly, a prior study provided evidence suggesting that LINC00152 (also known as CYTOR) is dysregulated in EC cells, which could position it as a potential biomarker for EC [18]. Moreover, LINC00152 has been implicated to confer L-OHP resistance in colon cancer, highlighting its potential involvement in drug resistance [19]. Therefore, we examine in this study the specific effect of LINC00152 on EMT and EC cell to L-OHP.

Enhancer zeste homolog 2 (EZH2), catalytic subunit of polycomb complex 2 (PRC2), which interacts with LINC00152 in lung adenocarcinoma [20] and the promotion of DNA methylation in ESCC cells by non-coding RNA POU3F3 [21], have drawn much attention in recent years due to their involvement in the development of human diseases, such as [22] lung cancer [23], gastric cancer [24], breast cancer [25], T-cell acute lymphoblastic leukemia [26], and pediatric soft tissue sarcomas [27]. Herein, we hypothesize that LINC00152 might also interact with EZH2 in EC cells.

EMT-induced transcriptional repressor, Zinc finger e-box binding homeobox 1 (ZEB1) is recognized as a stimulator towards imparting an aggressive, metastatic, and therapy-resistant cancer phenotype [28]. In addition, ZEB1 together with EZH2, is reported to play a key role in EMT in respiratory virus infection [29]. Based on our database findings and results from previous studies, we inferred that LINC00152 might regulate ZEB1 *via* interacting with EZH2 in EC. Therefore, we explored the regulatory relationship of the LINC00152 EZH2/ZEB1 axis and its involvement in EMT as well as resistance of EC cells to L-OHP, aiming to establish a new therapeutic channel for better treatment of EC.

## Materials and methods

### Ethics statement

The study protocol was approved by the Ethics Committee and Experimental Animal Ethics Committee of Cancer Hospital of Shantou University Medical College. All individuals signed informed written consent documents. Extensive efforts were made to ensure minimal suffering of the animals used in the study.

### Study subjects

In this study, EC tissues and adjacent normal tissues were collected from 76 EC patients in Cancer Hospital of Shantou University Medical College from 2016 to 2018. None of those patients had received radiotherapy and chemotherapy before surgery.

### Cell culture

The normal human esophageal epithelial cell line Het-1A and EC cell lines Kyse-30, Kyse-70, Kyse-150, TE-1 and TE-6 were purchased from Tumor Cell Bank of the Chinese Academy of Medical Science (Shanghai, China). All these cell lines were cultured in Roswell Park Memorial Institute (RPMI)-1640 medium (61,870,044, Gibco, Carlsbad, CA, USA) containing 10% fetal bovine serum (FBS, Gibco, Carlsbad, CA, USA), 50 U/mL penicillin and 50 µg/mL streptomycin (15,070,063, Gibco, Carlsbad, CA, USA) in a 37 °C incubator with 5% CO_2_. Oxaliplatin (L-OHP) was dissolved in phosphate buffered saline (PBS) to prepare solutions at different concentrations (0.5, 1, 2.5, 5.0 and 10.0 µM, which were stored at 4 °C until use.

### Cell counting kit-8 (CCK-8) assay

Cell viability was assessed with a CCK-8 kit (GK10001, GLPBIO, Shanghai, China) following the manufacturer’s protocol. After adding 100 µL of CCK-8 solution in each well, cells were incubated at room temperature for 2 h. The cell viability curve was plotted using optical density (OD) value measured at 460 nm at each time point. Experiments were independently repeated in triplicate in duplicate.

### Transient transfection

Kyse-150 and TE-1 cells were Three anti-LINC00152 siRNA constructs (named si-LINC00152-1, si-LINC00152-2, and si-LINC00152-3), anti-EZH2 siRNA (si-EZH2), anti-ZEB1 siRNA (si-ZEB1), LINC00152 expression vector (oe-LINC00152), EZH2 expression vector (oe-EZH2), ZEB1 expression vector (oe-ZEB1), and their negative controls (NC) were delivered into Kyse-150 and TE-1 cells, respectively, by using Lipofectamine 2000 reagents according to the manufacturer’s protocols (Invitrogen, Carlsbad, CA, USA). All siRNA constructs and expression vectors were purchased from Shanghai Sangon Biotech company (Shanghai, China), who also generated primer sequences and plasmid construction for siRNA sequences, as shown in Table 1. 48 h after transfection, cells were collected for further analysis. The experiment was repeated in triplicate.

**Table 1:** siRNA sequences

siRNA	Sequence (5′-3′)
si-NC	F: 5′-UUCUCCGAACGUGUCACGUTT-3′
	R: 5’-ACGUGACACG UUCGGAGAATT-3′
si-LINC00152-1	F: 5′-GGAAUGCAGCUGAAAGAUUTT-3′
	R: 5′-AAUCUUUCAGCUGCAUUCCTT-3′
si-LINC00152-2	F: 5′-GGUGGUCUGCCUGUGAUAUTT-3′
	R: 5′-AUAUCACAGGCAGACCACCTT-3′
si-LINC00152-3	F: 5′-TGCCGGAATGCAGCTGAAAGATTTCAA-3′
	R: 5′-TCGAGAAAAAAGCCGAATGGAATGCAT-3′
si-EZH2	F: 5′-AUCAGCUCGUCUGAACCUCUU-3′
	R: 5′-AAGAGGUUCAGACGAGCUGAU-3′
si-ZEB1	F: 5′-CCUAGUCAGCCACCUUUAATT-3′
	R: 5′-UUA AAGGUGGCUGACUAGGTT-3′

*RT-qPCR* reverse transcription quantitative polymerase chain reaction, *EZH2 *Zeste Homologue 2, *ZEB1* zinc finger e-box binding homeobox 1, *F* forward, *R* reverse

### Reverse transcription quantitative polymerase chain reaction (RT-qPCR)

Total RNA from tissues or cells was extracted using Trizol reagent (Thermo Fisher Scientific, Waltham, MA, USA). The synthesis of primer was conducted by Sangon Biotech Co., Ltd, (Shanghai, China) (Table 2). Reverse transcription was performed according to instructions of High-Capacity cDNA Reverse Transcription Kit (4,368,813, Thermo Fisher Scientific, Waltham, MA, USA) to generate cDNA. Real-Time Quantitative fluorogenic PCR assay was developed according to instructions of PCR kit (11,732,020, Thermo Fisher Scientific, Waltham, MA, USA). The relative expression level of target genes was measured by 2^−ΔΔCt^ method normalized to glyceraldehyde-3-phosphate dehydrogenase (GAPDH) mRNA expression.

**Table 2:** Primer sequences for RT-qPCR

Gene	Sequence
LINC00152	F: 5′-CTCCAGCACCTCTACCTGTTG-3′
	R: 5′-GGACAAGGGATTAAGACACACA-3′
EZH2	F: 5′-GGCTCCTCTAACCATGTTTACAACT-3′
	R: 5′-AGCGGTTTTGACACTCTGAACTAC-3′
ZEB1	F: 5′-ACTCTGATTCTACACCGC-3′
	R: 5′-TGTCACATTGATAGGGCTT-3′
GAPDH	F: 5′-GCACCGTCAAGGCTGAGAAC-3′
	R: 5′-ATGGTGGTGAAGACGCCAGT-3′

*RT-qPCR* reverse transcription quantitative polymerase chain reaction, *EZH2* Zeste Homologue 2, *ZEB1* zinc finger e-box binding homeobox 1, *GAPDH* glyceraldehyde-3-phosphate dehydrogenase, *F *forward, *R* reverse

### RNA immunoprecipitation (RIP) assay

The binding of LINC00152 to EZH2 protein was measured using a RIP kit (17–701, Millipore Corp, Billerica, MA, USA) according to the manufacturer’s instructions. Upon cell confluence in six-well plate reaching about 80–90%, cells in each well were lysed with an equal volume of RIPA lysis buffer (P0013B, Beyotime Institute of Biotechnology, Shanghai, China) for 5 min in an ice-bath and then centrifuged at 14,000 rpm for 10 min at 4 °C. A certain portion of supernatant was removed to serve as an input, and the remainder was co-precipitated by incubation with antibody. In brief, 50 µL of magnetic beads in each coprecipitation reaction system were resuspended in 100 µL of RIP wash buffer and incubated with 5 µg of corresponding antibody. Subsequently, the magnetic bead-antibody complex was washed and resuspended in 900 µL of RIP wash buffer and then incubated with 100 µL of cell extract overnight at 4 °C, whereupon it was placed on a magnetic stand to collect the magnetic bead-protein complex. Afterwards, samples and inputs were separately treated with proteinase K to extract RNA for subsequent PCR detection of LINC00152. The antibodies used for RIP were EZH2 (1:100, ab186006, Abcam, Cambridge, UK), SUZ12 (1:100, ab12073, Abcam, Cambridge, UK) and EED (SC-293,203, 1:100, Santa Cruz Biotechnology, Santa Cruz, CA, USA) with IgG (1:100, ab200699, Abcam, Cambridge, UK) as a NC. The experiment was repeated in triplicate.

### RNA pull-down

Cells were transfected with biotinylated LINC00152-Sense strand and LINC00152-Antisense strand (50 nM for each). At 48 h post transfection, cells were washed, vortexed and incubated with specific cell lysis buffer (Ambion, Austin, TX, USA) for 10 min, taking a 50 mL portion of cell lysate as a control. The remaining lysate was incubated with M-280 streptavidin magnetic beads (Sigma, St. Louis, MO, USA) pre-coated with RNase-free and yeast tRNA (Sigma, St. Louis, MO, USA) for 3 h at 4 °C, followed by two washes with cold lysis buffer, three washes with low salt buffer, and one wash with high salt buffer. Afterwards, total protein was extracted with high-efficiency RIPA lysis buffer and then the expression of EZH2 (1:100, ab186006, Abcam, Cambridge, UK) was determined by Western blot analysis. The experiment was repeated in triplicate.

### Fluorescence in situ hybridization (FISH)

The localization of LINC00152 in cells was examined using a FISH Kit (F32956, Thermo Fisher Scientific, Waltham, MA, USA) with digoxigenin-labeled LINC00152 probe, purchased from Sigma-Aldrich, St. Louis, MO, USA. Cell nuclei were stained with 4′, 6-diamidino-2-phenylindole (DAPI, D9542, Sigma-Aldrich, St. Louis, MO, USA) for 10 min. A laser confocal scanning microscope (FV1000, Olympus, Tokyo, Japan) was employed to take photos of fluorescence staining, and experiment was repeated in triplicate.

### Western blot analysis

Total protein was extracted using a protein extraction kit (BC3640, Beijing Solarbio Science and Technology Co., Ltd., Beijing, China), and was then quantified using a bicinchoninic acid (BCA) protein assay kit (20201ES76, Yeasen Biotech Co., Ltd., Shanghai, China). The extracted proteins were separated by 10% sodium dodecyl sulfate-polyacrylamide gel electrophoresis (SDS-PAGE), and then transferred onto a polyvinylidene fluoride (PVDF; Bio-Rad Laboratories, Hercules, CA, USA) membrane. Subsequently, the PVDF membrane were incubated overnight at 4 °C with diluted primary monoclonal antibodies against the target proteins GAPDH (ab181602, 1:5000), E-cadherin (ab1416, 1:1000), vimentin (ab193555, 1:1000), cleaved poly adenosine diphosphate-ribose polymerase (PARP; ab32064, 1:1000), cleaved Caspase3 (ab2302, 1:1000), EZH2 (ab186006, 1:1000), ZEB1 (ab228986, 1:1000), and PARP. Afterwards, the membrane was blotted with corresponding secondary antibody conjugated to horseradish peroxidase, namely, goat anti-rabbit antibody to immunoglobulin G (IgG; ab6721, 1:2000) or rabbit anti-mouse (ab6728, 1:2000) for 1 h at 4 °C. All antibodies were purchased from Abcam (Cambridge, UK). After washed with PBS with 5 min for each time, membranes were incubated with enhanced chemiluminescence (ECL) (Pierce, Waltham, MA, USA) for about 1 min and were then semi-quantified using an Image Quant LAS-4000 image reader (GE Healthcare, Piscataway, NJ, USA). The analysis of protein expression levels was obtained as the ratio of gray values between proteins of interest and internal reference standard GAPDH.

### Chromatin immunoprecipitation (ChIP)

ChIP experiments were performed using EZ-Magna ChIP kit (EMD Millipore Corp., Billerica, MA, USA) according to protocol provided by manufacturer. MSCs cells were fixed with 1% paraformaldehyde and incubated with glycine for 10 min to crosslink DNA and protein. Then, cells were lysed with lysis buffer and sonicated to produce 200–300 bp chromatin fragments. Next, lysate was immunoprecipitated with magnetic protein A beads, which were bound to antibodies targeting the proteins of interest, namely, EZH2 (ab191250, 1:20, Abcam, Cambridge, UK) and methylation of lysine 27 on histone H3 (H3K27me3; ab6002, 1:20, Abcam, Cambridge, UK). Meanwhile, NC was added with IgG (ab2410, 1:20, Abcam, Cambridge, UK). Finally, precipitated ZEB1 was analyzed by RT-qPCR assay. ZEB CHIP primer was (F: 5’-AGGCGTGGGACTGATGGTAG-3’, R: 5’-ATTCTCCCTGTACCCTGTGC-3’).

### Xenograft tumor in nude mice

Twenty-four specific pathogen-free (SPF) female BALB/C nude mice aged 4 weeks and weighing 18–22 g purchased from Shanghai SLAC Laboratory Animal Co., Ltd. (Shanghai, China) were employed for animal experiments. EC cells Kyse-150 were cultured in a culture dish. When cell confluence reached 90%, cells were detached with a conventional trypsin buffer and rinsed twice with Dulbecco’s modified Eagle’s medium (DMEM) to prepare a single cell suspension. The cell suspension was centrifuged at 1500 rpm for 3 min to remove the supernatant, followed by addition of DMEM. The number of blood cells was counted under the microscope and cell concentration was adjusted to 5 × 10^7^ cells/mL. Cells were preserved on ice for 1 h and then inoculated subcutaneously into the nude mice. About 10 days later, solid tumors had formed; upon attaining a tumor volume of about 100 mm^3^, groups of mice were injected intravenously with (1) oe-NC and si-NC, (2) oe-NC and si-NC plus L-OHP, (3) oe-LINC0015 and si-NC plus L-OHP, or (4) both oe-LINC0015 and si-ZEB1 plus L-OHP to explore how LINC00152 regulates the EZH2/ZEB1 axis in the context of EC cell drug resistance. 25 days after treatment, the mice were euthanized, and the tumors were extracted from the mice, washed in normal saline, and photographed.

### Statistical analysis

The data were processed using SPSS 21.0 statistical software (IBM Corp, Armonk, NY, USA). Measurement data were expressed as mean ± standard deviation. Paired data in compliance with normal distribution and homogeneity between cancer tissues and adjacent tissues were compared using a paired *t*-test. Comparisons among multiple groups were conducted by one-way analysis of variance (ANOVA) with Tukey’s *post hoc* test. Data at different time points and different concentrations were compared by repeated measures ANOVA. A value of *p* < 0.05 indicated significant difference.

## Results

### **LINC00152 is highly expressed in EC tissues and cells and enhances EC cell resistance to L-OHP** 

**Fig. 1:**
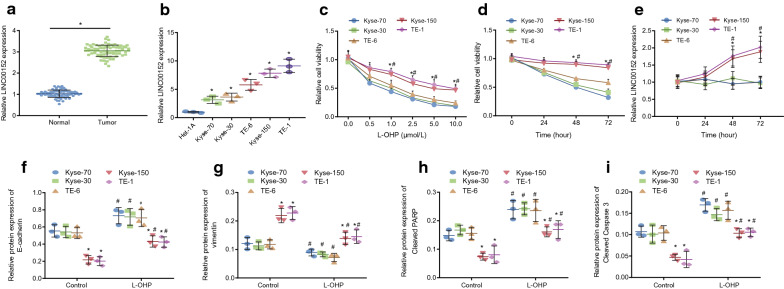
upregulated LINC00152 is associated with EC cell resistance to L-OHP. **a** the expression of LINC00152 in EC tissues and normal adjacent tissues detected by RT-qPCR assay. * *p* < 0.05 vs. normal adjacent tissues (analyzed by paired *t*-test). **b** the expression of LINC00152 in Kyse-30, Kyse-70, Kyse-150, TE-1 and TE-6 cell lines detected by RT-qPCR assay. * *p* < 0.05 vs. Het-1A cells (analyzed by one-way ANOVA with Tukey’s *post hoc* test).** c** cell survival rate after 72 h of treatment with different doses of L-OHP (0, 0.5, 1.0, 2.5, 5.0, 10.0 µM) detected by CCK-8 assay.** d** cell survival rate after 0, 24, 48 and 72 h of treatment with 10.0 µM of L-OHP detected by CCK-8 assay.** e**, the expression of LINC00152 after 0, 24, 48 and 72 h of treatment with 10.0 µM of L-OHP detected by RT-qPCR assay. * indicates *p* < 0.05 when Kyse-150 vs.Kyse-30, Kyse-70, and TE-6, respectively; # *p* < 0.05 when TE-1 vs. Kyse-30, Kyse-70, and TE-6, respectively (analyzed by one-way ANOVA with Tukey’s *post hoc* test or repeated measures ANOVA with Bonferroni corrections).** f–i**, Western blot analysis of E-cadherin, vimentin, cleaved PARP, and cleaved Caspase 3 in five EC cells with or without after 72-hour treatment of 10.0 µM L-OHP. * indicates *p* < 0.05 vs. parental or L-OHP-treated Kyse-30, Kyse-70, and TE-6, respectively; # indicates *p* < 0.05 vs. corresponding parental EC cells (analyzed by one-way ANOVA with Tukey’s *post hoc* test or repeated measures ANOVA with Bonferroni corrections). Data are presented as mean ± standard deviation of three technical replicates

It has been reported that LINC00152 is highly expressed in EC, which might induce the occurrence of EC [18]. Besides, LINC00152 contributes to colon cancer resistance to L-OHP [19]. We now sought to investigate whether LINC00152 augmented EC cells to develop L-OHP resistance. First, the findings of the RT-qPCR assay indicated notably increased expression level of LINC00152 in EC tissues compared to normal adjacent tissues (Fig. 1a; *p* < 0.05). Accordingly, compared with Het-1A cell line, the expression level of LINC00152 was notably elevated in Kyse-30, Kyse-70, Kyse-150, TE-1 and TE-6 cell lines, among which, expression of LINC00152 was highest in Kyse-150 and TE-1 cell lines (Fig. 1b; *p* < 0.05).

The resistance to L-OHP was then tested among the five different EC cell lines. Firs, different doses of L-OHP (0.0, 0.5, 1.0, 2.5, 5.0, 10.0 µM) was added to theseEC cell lines followed by culture for 72 h. CCK-8 assay results indicated a L-OHP dose-dependent decrease of survival rate of all cells, with survival rate of Kyse-150 and TE-1 cells higher than those of other 3 cells at 1.0, 2.5, 5.0, and 10 µM L-OHP concentrations (Fig. 1c; *p* < 0.05). Next, the 5 EC cell cultures were all treated with 10 µM L-OHP and their cell viability and expression of LINC00152 were detected by CCK-8 and RT-qPCR assay, respectively, at 0, 24, 48, and 72 hours in culture. Again, the aforementioned data illustrated that Kyse-150 and TE-1 cells had higher survival rate than other three cell lines at 48 and 72 h of treatment (Fig. 1d; *p* < 0.05). Meanwhile, expression of LINC00152 was notably elevated in Kyse-150 and TE-1 cells with treatment for 48 and 72 h (Fig. 1e; *p* < 0.05).

To detect resistance to L-OHP and EMT of different EC cell lines, different EC cell lines were treated with 10 µ L-OHP for 72 h. Western blot analysis suggested that, compared with cells treated with equal PBS, protein expression of cleaved PARP, cleaved Caspase 3 and E-cadherin was notably elevated in the Kyse-30, Kyse-70, TE-6, Kyse-150 and TE-1 cells, which were resistant to L-OHP (*p* < 0.05), while expression of vimentin protein notably diminished (*p* < 0.05). Compared with L-OHP-resistant Kyse-30, Kyse-70, TE-6 cells, expression of cleaved PARP, cleaved Caspase 3 and E-cadherin protein were diminished in L-OHP-resistant Kyse-150 and TE-1 cells (*p* < 0.05), and expression of vimentin protein elevated (Fig. 1f–i; *p* < 0.05).

These results indicated that different EC cell lines exhibited different degrees of sensitivity to L-OHP as evidenced by their cell survival rates, and that the lines with highest resistance (Kyse-150 and TE-1 cell lines) had the highest expression of LINC00152 and could affect EMT. Due to their higher drug resistance to L-OHP, Kyse-150 and TE-1 cell lines were selected for subsequent experiments.

#### Downregulation of LINC00152 reduces EMT and EC cell resistance to L-OHP

**Fig. 2:**
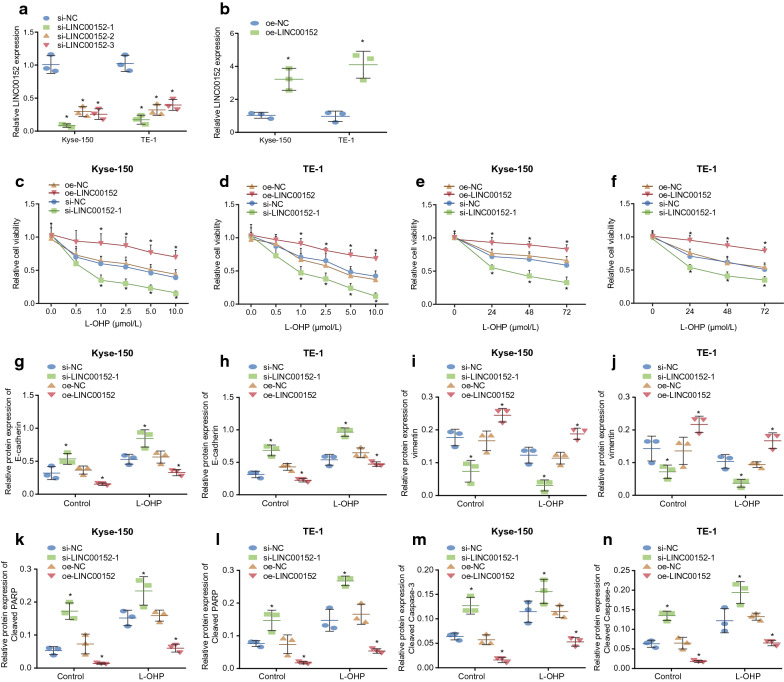
Silencing of LINC00152 attenuated EMT and EC cell resistance to L-OHP. **a–b** the expression of LINC00152 after overexpressing or knocking down LINC00152 in Kyse-150 and TE-1 cells detected by RT-qPCR assay. **c–d** cell survival rate after 72 h of treatment with different concentrations of L-OHP (0, 0.5, 1.0, 2.5, 5.0, 10.0 µM) in Kyse-150 and TE-1 cells detected by CCK-8 assay. **e–f** cell survival rate after 0, 24, 48 and 72 h of treatment with 10.0 µM of L-OHP in Kyse-150 and TE-1 cells detected by CCK-8 assay.** g–n** the protein expression of E-cadherin, cleaved PARP and cleaved Caspase 3 in Kyse-150 and TE-1 cells after 72 h of treatment with 10.0 µmol/L of L-OHP determined by Western blot analysis. * *p* < 0.05 vs. the oe-NC group or the si-NC group. Data in compliance with normal distribution and homogeneity were compared using unpaired *t*-test. Comparisons among multiple groups were conducted by ANOVA with Tukey’s post hoc test. Data at different time points and different concentrations were compared by repeated measures ANOVA. Data are presented as mean ± standard deviation of three technical replicates

The aforementioned evidence shows that LINC00152 might be a factor in EMT and resistance to L-OHP in EC cells. To verify effect of LINC00152 on EC cells, we overexpressed and knocked down LINC00152 separately in Kyse-150 and TE-1 cells. Results of RT-qPCR revealed that compared with si-NC, all anti-LINC00152 siRNA constructs (si-LINC00152-1, si-LINC00152-2, and si-LINC00152-3) effectively knocked down LINC00152. As shown in Fig. 2a, si-LINC00152-1 was selected to knockdown LINC00152-1 for subsequent experiments. Likewise, compared with oe-NC, expression level of LINC00152 in oe-LINC00152 group was notably elevated (Fig. 2b; *p* < 0.05). After LINC00152 was overexpressed or knocked down in Kyse-150 and TE-1 cells, the cells were treated with different doses of L-OHP for 72 h. CCK-8 assay then showed that, compared with cells transfected with oe-NC, the survival rate of oe-LINC00152 transfected cells was notably elevated (*p* < 0.05). Accordingly, compared with si-NC, survival rate of si-LINC00152-1-treated cells was notably diminished at doses of 1.0, 2.5, 5.0, and 10 µM (Fig. 2c–d; *p* < 0.05). Setting the dose of L-OHP treatment to 10 µM, the CCK-8 assay suggested that, compared with cells transfected with oe-NC, survival rate in oe-LINC00152 notably elevated at 24, 48 and 72 h (*p* < 0.05), while compared with si-NC treatment, survival rate following si-LINC00152-1 treatment notably diminished (Fig. 2e–f; *p* < 0.05).

Next, we investigated whether downregulation of LINC00152 could affect EMT and drug resistance of EC cells. After overexpressing or knocking down LINC00152 in Kyse-150 and TE-1 cells, these cells were treated with 10 µM L-OHP for 72 h. Results of Western blot analysis suggested that after the L-OHP treatment, there was notably elevated protein expression of E-cadherin, cleaved PARP and cleaved Caspase 3 in si-LINC00152-1 transfected cells compared with the si-NC group (*p* < 0.05), while expression of vimentin markedly diminished (*p* < 0.05). On the other hand, compared with cells transfected with oe-NC, expression level of E-cadherin, cleaved PARP and cleaved Caspase 3 protein was notably lower in oe-LINC00152 transfected EC cells (*p* < 0.05), while expression of vimentin was notably elevated (Fig. 2g-n; *p* < 0.05). These results suggested that downregulation of LINC00152 was able to decrease EMT and drug resistance in EC cells.

### LINC00152 promotes EC cell resistance to L-OHP and EMT through recruitment of EZH2

**Fig. 3:**
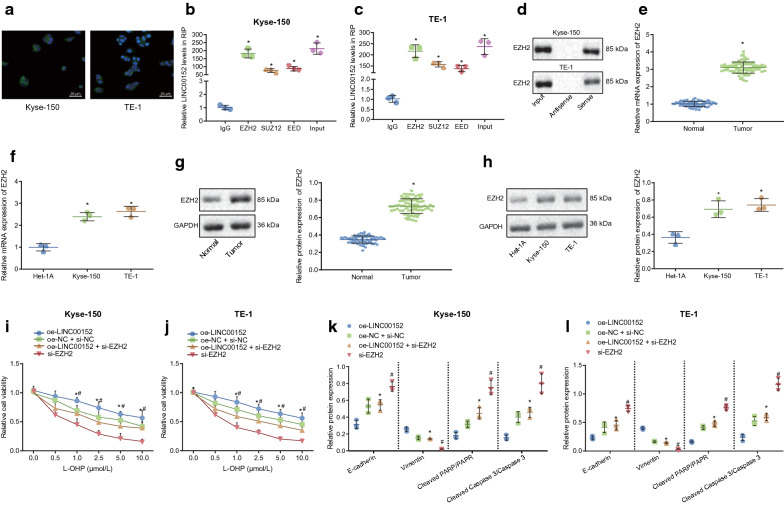
LINC00152 promotes EMT and EC cell resistance to L-OHP by interacting with EZH2.** a** localization of LINC00152 in cytoplasm and nucleus in Kyse-150 and TE-1 cells determined by FISH assay (400 ×).** b–c** the interaction between LINC00152 and PRC2 in Kyse-150 and TE-1 cells determined by RIP assay.** d** binding of LINC00152 and PRC2 detected by RNA pull-down assay.** e** the mRNA expression of EZH2 in EC issues detected by RT-qPCR assay.** f** the mRNA expression of EZH2 in in Kyse-150 and TE-1 cells detected by RT-qPCR assay.** g–h** the protein expression of EZH2 in Kyse-150 and TE-1 cells measured by western blot analysis.** i**, ** J** cell survival rate after 72 h of treatment with different concentrations of L-OHP (0, 0.5, 1.0, 2.5, 5.0, 10.0 µM) in Kyse-150 and TE-1 cells detected by CCK-8 assay.** k**,** l** the protein expression of E-cadherin, vimentin, cleaved PARP/PARP and cleaved Caspase 3/Caspase 3 after 72 h of treatment with 10.0 µM of L-OHP, determined by Western blot analysis. In panel** b–c**, * *p* < 0.05 vs. IgG. In panel **e–g** * *p* < 0.05 vs. normal adjacent tissues or Het-1A cells. * *p* < 0.05 vs. cells transfected with oe-LINC00152, # *p* < 0.05 vs. cells transfected with oe-LINC00152 and si-EZH2. Paired data in compliance with normal distribution and homogeneity between cancer tissues and adjacent tissues were compared using paired *t*-test. Comparisons among multiple groups were conducted by ANOVA with Tukey’s post hoc test. Data at different time points and different concentrations were compared by repeated measures ANOVA. Data are presented as mean ± standard deviation of three technical replicates

A previous study suggested that LINC00152 can interact with EZH2 to regulate expression of their downstream genes [20]. Here, FISH assay results suggested LINC00152 was expressed and localized in the nucleus and cytoplasm in Kyse-150 and TE-1 cells (Fig. 3a). It was also previously reported that lncRNA MALAT1 releases EZH2 by binding to the PRC2 complex, a main component of which is EZH2, which hinders transcriptional inhibition of downstream genes, thereby promoting expression of downstream genes [30]. Thus, we inferred that LINC00152 would release EZH2 by binding to a complex of PRC2, thus reducing trimethylation of h3k27 and blocking transcriptional inhibition of the zeb1 gene, consequently promoting the expression of zeb1. to verify whether linc00152 bound to prc2, interactions between linc00152 and ezh2, suz12 along with eed were assessed by rip assay. analysis of rt-qpcr revealed that the amounts of linc00152 pulled by ezh2, sUZ12 and EED were notably higher than with IgG (Fig. 3b-c; *p* < 0.05). Next, RNA pull-down assay was used to verify further an interaction between LINC00152 and EZH2. The sense and antisense chains of LINC00152 were incubated with cell extracts, and the EZH2 protein content was measured by Western blot analysis. The aforementioned data illustrated that EZH2 protein interacted with the sense chain of LINC00152 (Fig. 3d). Next, we investigated whether EZH2 mediated effects of LINC00152 on the regulation of EMT and drug resistance of EC cells. First, RT-qPCR results displayed that mRNA expression of EZH2 in EC tissues, Kyse-150 and TE-1 cells were notably higher than those in normal adjacent tissues and Het-1A cells (Fig. 3e-f; *p* < 0.05). Then, results of Western blot analysis suggested that protein expression of EZH2 in EC tissues, Kyse-150 and TE-1 cells was also notably higher than that in normal adjacent tissues and Het-1A, respectively (Fig. 3g-h; *p* < 0.05).

Results of the CCK-8 assay suggested that, compared with cells transfected with oe-LINC00152, survival rate in cells transfected with oe-LINC00152 and si-EZH2 was notably lower after treatments of different doses of L-OHP in Kyse-150 and TE-1 cells for 72 h (*p* < 0.05). Compared with cells transfected with oe-LINC00152 and si-EZH2, survival rate of cells transfected with si-EZH2 was also notably lower (Fig. 3i–j; *p* < 0.05). Moreover, Western blot analysis suggested that after 10 µM L-OHP treatment for 72 h, protein expression of E-cadherin and cleaved PARP/PARP and cleaved Caspase 3/Caspase 3 in cells transfected with oe-LINC00152 and si-EZH2 was notably elevated compared with that in oe-LINC00152 transfected-cells (*p* < 0.05), while protein expression of vimentin was notably diminished (*p* < 0.05). Compared with those in oe-LINC00152 and si-EZH2 transfected-cells, protein expression of E-cadherin, cleaved PARP/PARP and cleaved Caspase 3/Caspase 3 in si-EZH2 transfected-cells was notably enhanced (*p* < 0.05), while protein expression of vimentin was notably diminished (Fig. 3k–l) (*p* < 0.05). The above results indicated that LINC00152 upregulates EMT and drug resistance through positively regulating EZH2.

### LINC00152 increases expression of ZEB1 through EZH2 in EC cells

**Fig. 4:**
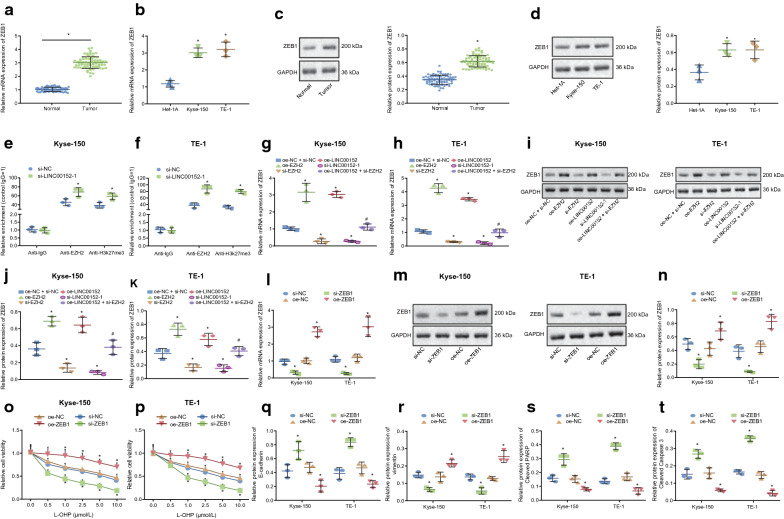
LINC00152 increases the expression of ZEB1 through interaction with EZH2 in EC cells.** a–b** the mRNA expression of ZEB1 in EC tissues, Kyse-150 and TE-1 cells detected by RT-PCR assay.** c** the protein expression of ZEB1 in EC tissues, Kyse-150 and TE-1 cells determined by Western blot analysis. **d** Statistical histogram of panel C. **e–f** the enrichment of EZH2 and H3K27me3 in ZEB1 promoter region in Kyse-150 cells and TE-1 cells determined by ChIP assay. **g–h** the mRNA expression of ZEB1 in transfected Kyse-150 and TE-1 cells. **i** the protein expression of ZEB1 in transfected Kyse-150 and TE-1 cells; **j–k** Statistical histogram of panel I. **l** the mRNA expression of ZEB1 in Kyse-150 and TE-1 cells transfected with oe-ZEB1 or si-ZEB1. **m** the protein expression of ZEB1 in Kyse-150 and TE-1 cells transfected with oe-ZEB1 or si-ZEB1, **n** Statistical histogram of panel M. **o–p** cell survival rate after 72 h of treatment with different doses of L-OHP (0, 0.5, 1.0, 2.5, 5.0, 10.0 µM) in Kyse-150 and TE-1 cells detected by CCK-8 assay. **q–t** the protein expression of E-cadherin, vimentin, cleaved PARP and cleaved Caspase 3 protein after 72 h of treatment with 10.0 µM of L-OHP determined by Western blot analysis. In panel **a–d**, * *p* < 0.05 vs. normal adjacent tissues or Het-1A cells. In panel **e-f**, * *p* < 0.05 vs. the si-NC group. In** g–k** * *p* < 0.05 vs. the oe-NC + si-NC group, # *p* < 0.05 vs. the oe-LINC00152 group. In panel** l–t**, * *p* < 0.05 vs. the oe-NC group. # *p* < 0.05 vs. the oe-NC group. Paired data in compliance with normal distribution and homogeneity between cancer tissues and adjacent tissues were compared using paired *t*-test. Comparisons among multiple groups were conducted by ANOVA with Tukey’s *post hoc* test. Data at different time points and different concentrations were compared by repeated measures ANOVA. Data are presented as mean ± standard deviation of three technical replicates

EZH2 was highly expressed in EC tissues as well as Kyse-150 and TE-1 cell lines. It has been reported that overexpression of EZH2 can promote expression of ZEB1 [31]. Here, we analyzed the expression level of ZEB1 in EC tissues, Kyse-150 and TE-1 cells by RT-qPCR (Fig. 4a–b) and Western blot analysis (Fig. 4c–d). The results illustrated that mRNA level and protein expression level of ZEB1 in EC tissues, Kyse-150 and TE-1 cells were notably higher than those in normal adjacent tissues and Het-1A cells (*p* < 0.05).

In addition, we tested whether EZH2 could also increase expression of ZEB1 in EC cells. Results of ChIP assay suggested that treatment with 10.0 µM L-OHP for 72 h, there was significant enrichment of EZH2 and H3k27me3 in the promoter region of ZEB1 gene in si-LINC00152-1 transfected Kyse-150 and TE-1 cells compared with si-NC transfected-cells (Fig. 4e–f; *p* < 0.05). After different transfections of Kyse-150 and TE-1 cells, results of RT-qPCR assay (Fig. 4g–h) and Western blot analysis (Fig. 4i–k) displayed that mRNA and protein expression of ZEB1 in si-EZH2 and si-LINC00152-1 transfected-cells were notably lower than those in oe-NC and si-NC transfected-cells (*p* < 0.05). On the other hand, mRNA and protein expression of ZEB1 in oe-EZH2 and oe-LINC00152 transfected-cells were notably higher. Compared with oe-LINC00152 transfected-cells, mRNA and protein expression of ZEB1 were notably diminished in oe-LINC00152 and si-EZH2 transfected cells. All these results demonstrated that EZH2 upregulated expression of ZEB1 in EC cells.

Further, we tested whether downregulation of ZEB1 could delay EMT and drug resistance of EC cells to L-OHP. Results of RT-qPCR (Fig. 4l) and Western blot analysis (Fig. 4m–n) suggested mRNA and protein expression of ZEB1 in si-ZEB1 transfected-cells was notably lower than that in si-NC transfected-cells (*p* < 0.05). Conversely, mRNA and protein expression of ZEB1 in oe-ZEB1 transfected-cells was notably higher than in oe-NC transfected-cells. The CCK-8 assay results suggested that, after treatment with different doses of L-OHP for 72 h, the survival rate in oe-ZEB1 transfected Kyse-150 and TE-1 cells was notably elevated compared with oe-NC transfected cells (*p* < 0.05), and notably diminished in si-ZEB1 transfected-cells compared with si-NC transfected-cells (Fig. 4o–p) (*p* < 0.05). Then, we determined expression of E-cadherin, vimentin, cleaved PARP and cleaved Caspase 3 in Kyse-150 and TE-1 cells treated with 10 µM L-OHP for 72 h. Results indicated that, compared with si-NC transfected cells, protein expression of E-cadherin, cleaved PARP and cleaved Caspase 3 in si-ZEB1 transfected-cells was notably elevated (*p* < 0.05), and vimentin expression was notably diminished. On the other hand, compared with oe-NC transfected-cells, protein expression of E-cadherin, cleaved PARP and cleaved Caspase 3 was notably diminished, and vimentin expression was notably elevated (Fig. 4q–t; *p* < 0.05). The above results indicated that LINC00152 upregulated expression of ZEB1 through itsinteraction with EZH2, thus promoting EMT and drug resistance of EC cells to L-OHP.

### **Downregulation of ZEB1 reverses promotive effect of LINC00152 on EMT and EC cell resistance to L-OHP** 

**Fig. 5:**
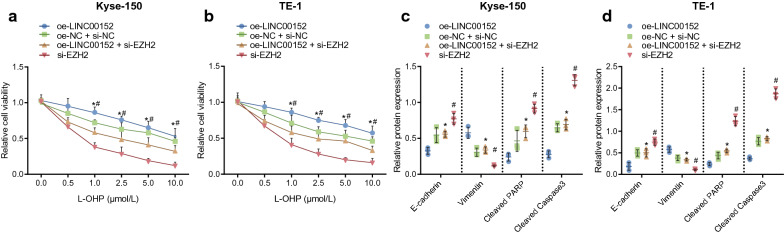
LINC00152 promotes EMT and EC cell resistance to L-OHP by upregulating ZEB1. **a–b** cell survival rate after 72 h of treatment with different concentrations of L-OHP (0, 0.5, 1.0, 2.5, 5.0, 10.0 µM) in Kyse-150 and TE-1 cells detected by CCK-8 assay. **c–d**, the expression of E-cadherin, vimentin, cleaved PARP/PARP and cleaved Caspase 3/Caspase 3 proteins in Kyse-150 (**c**) and TE-1 cells (**d**) after 72 h treatment with 10.0 µM of L-OHP determined by Western blot analysis. # *p* < 0.05 vs. the si-ZEB1 group. Comparisons among multiple groups were conducted by ANOVA with Tukey’s post hoc test. Data at different time points and different concentrations were compared by repeated measures ANOVA. Data are presented as mean ± standard deviation of three technical replicates

To verify further the effects of LINC00152 on regulation of EMT and drug resistance in EC cells was obtained through ZEB1, we transfected Kyse-150 and TE-1 cells with corresponding plasmids and then treated them with different doses of L-OHP for 72 h. The findings of the CCK-8 assay indicated a notably lower survival rate in oe-LINC00152 and si-ZEB1 transfected cells compared with oe-LINC00152 transfected cells, and that survival rate in si-ZEB1 transfected-cells was notably diminished compared with oe-LINC00152 and si-ZEB1 transfected cells (Fig. 5a–b). Then, findings of Western blot analysis for cells treated with 10 µM L-OHP for 72 h displayed that, compared with oe-LINC00152 transfected cells, protein expression of E-cadherin, cleaved PARP/PARP and cleaved Caspase 3/Caspase 3 in oe-LINC00152 and si-ZEB1 transfected cells was notably elevated, while vimentin was notably diminished. In comparison with oe-LINC00152 and si-ZEB1 transfected-cells, protein expression of E-cadherin, cleaved PARP/PARP and cleaved Caspase 3/Caspase 3 in si-ZEB1 transfected cells was notably elevated, and vimentin was notably attenuated (Fig. 5c–d). These results suggested that high expression of LINC00152 in EC cells promotes EMT and resistance of EC cells to L-OHP by upregulating ZEB1.

### **LINC00152 enhances EMT and EC cell resistance to L-OHP by regulating the EZH2/ZEB1 axis*in vivo***

**Fig. 6:**
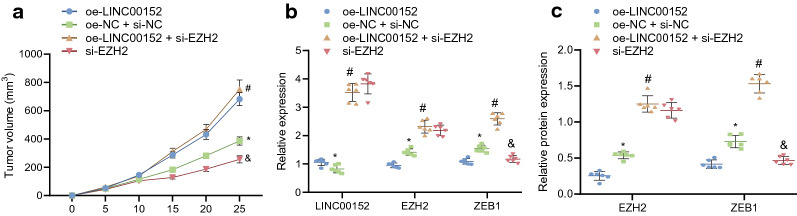
LINC00152 mediates the EZH2/ZEB1 axis to promote EMT and EC cell resistance to L-OHP. **a** tumor growth rate curves of nude mice xenografted with EC cells (n = 6). **b** the mRNA expression of LINC00152, EZH2, and ZEB1 in tumor tissues of nude mice xenografted with EC cells (n = 6) detected by RT-qPCR.** c** the protein expression of EZH2 and ZEB1 in tumor tissues of nude mice xenografted with EC cells (n = 6) detected by Western blot analysis. * *p* < 0.05 vs. The oe-NC + si-NC group; # *p* < 0.05 vs. the oe-NC + si-NC + L-OHP group; & *p* < 0.05 vs. the oe-LINC00152 + si-NC + L-OHP group. Data are presented as mean ± standard deviation of three technical replicates

A xenograft tumor model in nude mice was established to probe further how LINC00152 could promote EMT and resistance of EC cells to L-OHP treatment via the EZH2/ZEB1 axis. EC cells were subcutaneously inoculated into the nude mice to construct the EC tumor model, and the tumor-bearing mice were further treated with L-OHP combined with oe-LINC00152 or si-ZEB1. Results showed that, the tumor growth rate was increased in response to treatments with oe-LINC00152 plus L-OHP versus the treatments with oe-NC and si-NC plus L-OHP, thus revealing that overexpressed LINC00152 could elevate the resistance of EC. Furthermore, the treatments with si-ZEB1 plus L-OHP resulted in reduced tumor growth rate, indicating that ZEB1 knockdown could effectively attenuate the development of EC. Additionally, in comparison with the treatment of oe-LINC00152 and si-NC plus L-OHP, the treatment of oe-LINC00152 and si-ZEB1 plus L-OHP led to decreased tumor growth rate, indicating that silencing ZEB1 could inhibit the resistance in EC induced by LINC00152 overexpression (Fig. 6a) (*p* < 0.05).

RT-qPCR was conducted to measure the relative expression of LINC00152, EZH2, and ZEB1 while Western blot analysis was carried out to detect the protein expression of EZH2, and ZEB1. The results showed that the expression of LINC00152, EZH2, and ZEB1 was elevated in the tumors of mice treated with oe-LINC00152 and si-NC plus L-OHP versus the mice treated with oe-NC and si-NC plus L-OHP. The treatment with oe-LINC00152 and si-ZEB1 plus L-OHP decreased LINC00152, EZH2, and ZEB1 expression compared to the treatment with oe-LINC00152 and si-NC plus L-OHP (Fig. 6b, c) (*p* < 0.05).

These results revealed that, in the EC xenograft tumor model, treatment with oe-LINC00152 alone could induce greater drug resistance of EC cells, while the combination of oe-LINC00152 and si-ZEB1 increase the sensitivity of EC cells to L-OHP, thus reducing chemoresistance. We found that administration of si-ZEB1, by inducing overexpression of LINC00152, could reverse the effects of LINC00152 on EMT and resistance of EC cells to L-OHP.

## Discussion

Despite optimal current treatment, the survival rate of ESCC remains rather low due to its aggressive invasion and metastasis as well as the resistance to chemotherapy [1]. Emerging evidences have revealed that lncRNAs can be involved in tumorigenesis and development of EC [18, 32]. It has been reported that LINC00152 plays a crucial role in EMT and chemo-resistance in breast cancer cells [33]. Besides, LINC00152 was proven to contribute to resistance of colon cancer cell to L-OHP treatment [19]. Here, present study was conducted to explore the regulatory role of LINC00152 in L-OHP-induced EMT and resistance in EC, as well as testing the involvement of ZEB1.

The first finding in this study was that LINC00152 was highly expressed in EC cells, which is consistent with earlier results in gastric cancer [34] and ESCC [35]. Subsequently, we designed gain- and loss-of-function experiments to explore its effects on biological process of EC cells. Results indicated that LINC00152 contributed to enhanced EMT and resistance to L-OHP of EC cells, which concurred with findings in previous studies. For example, one previous study demonstrated that LINC00152 confers chemo-resistance of colorectal cancer cells to 5-FU treatment [36], and that LINC00152 is conductive to chemosensitivity of colon cancer cell to L-OHP [19]. Additionally, LINC00152 contributes to EMT in glioblastoma [37]. These literature results collectively suggested that LINC00152 played a promotive role in EMT and drug resistance of cancer cells. In contrast, inhibition of LINC00152 suppresses EMT and resistance of chemotherapy, as verified in breast cancer [33]. However, details of the regulatory mechanisms needed further clarification. Subsequently, our present investigation revealed that LINC00152 was capable of facilitating EMT and resistance of EC cells to L-OHP *via* interacting with EZH2, which is partially corroborating previous results that LINC00152 facilitates EMT in colon cancer by interaction with β-catenin.

EZH2 is an enhancer of H3K27me3, alteration of which are considered to be carcinogenic drivers of lymphoma and other malignancies [38]. This work confirmed that EZH2 was highly expressed in EC cells, concurring with its known expression pattern in ESCC [39]. More importantly, loss of EZH2 reversed induction of EMT and resistance of EC cells to L-OHP treatment, manifesting in reductions in vimentin, and increase expression of E-cadherin, cleaved PARP and cleaved Caspase 3. These results were partially consistent with a previous finding that LINC00152 diminished expression of E-cadherin *via* interacting with EZH2, thereby promoting EMT in hepatocellular carcinoma [40]. Additionally, a few studies have found that many lncRNAs exert pro-tumorigenic effects *via* interaction with EZH2. For instance, lncRNA H19 contributes to progression of tongue squamous cell carcinoma through interaction with EZH2 [41]. Also, lncRNA MALAT1 promotes EMT of EC through EZH2 cell [42]. Based on previous and present results, we conclude that LINC00152 contributes to EMT and resistance of EC cells to L-OHP *via* interaction with EZH2.

Further, we saw high expression in EC cells of ZEB1, which is proved to be an oncogene in various cancers, including colorectal cancer [40] and lung cancer [43], as well as ESCC [44]. Besides, lncRNA CASC15 was found to promote EMT in gastric cancer by targeting ZEB1 [45]. More importantly, it has been confirmed that lncRNA MALAT1 can release EZH2 by binding to PRC2 complex, of which EZH2 is a main component thus blocking transcriptional inhibition of downstream genes and consequently promoting the expression of HIV-1 in lung adenocarcinoma [28]. Additionally, lncRNA DANCR upregulates FBP1 to accelerate proliferation and migration in cholangiocarcinoma *via* interacting with EZH2 [46]. Similarly, LINC00152 regulates expression of downstream gene IL24 *via* interacting with EZH2 in lung adenocarcinoma [20]. In accord with those findings, our study suggests that LINC00152 enhanced EMT and resistance of EC cell to L-OHP by upregulating ZEB1 through interaction with EZH2.

## Conclusions

**Fig. 7:**
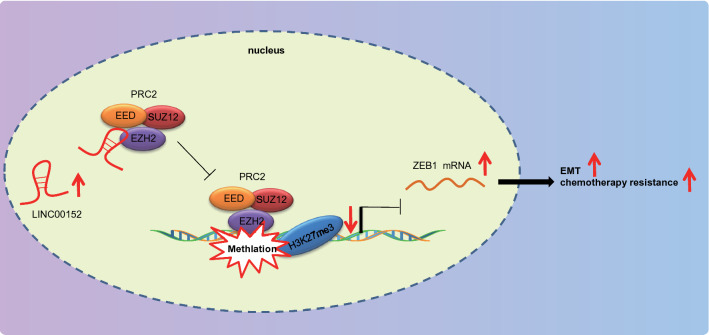
The mechanism graph of regulatory network and function of LINC00152/EZH2/ZEB1. The highly expressed LINC00152 in EC could bind to the PRC2 protein complex (EZH2) to hinder the transcriptional inhibition of the downstream gene ZEB1 and increase the expression of ZEB1 through interaction with EZH2. The overexpression of ZEB1, on the one hand, increases the vimentin (interstitial protein marker) expression and decreases E-cadherin (epithelial protein marker) expression, thereby accelerating EMT; meanwhile, LINC00152 reduces the cleaved PARP and cleaved Caspase 3 expression, resulting in EC cell resistance to L-OHP

To conclude, present findings collectively suggest that LINC00152 upregulated ZEB1 to enhance EC cell resistance to L-OHP and concomitant EMT through interaction with EZH2 (Fig. 7). Furthermore, silencing of LINC00152 led to impeded EMT and EC cell resistance to L-OHP. Thus, the present study contributes to identifying the role of LINC00152 in EC, offering a potential novel therapeutic target to repress EMT and resistance of EC cells to L-OHP.

## Limitation statement

It is possible that cancer cell biology and signaling pathways will differ from that in tumors *in vivo*, which is the limitation of all the cell-line-based research. In this study, EC tissues were collected only for determining the expression of LINC00152. We did not document differences in the expression of LINC00152 in relation to response to L-OHP treatment in the patients.

## Data Availability

The datasets used and/or analysed during the current study are available from the corresponding author on reasonable request.

## Abbreviations

EC: Esophageal cancer
EMT: Epithelial-mesenchymal transition
L-OHP: Oxaliplatin
EZH2: Enhancer zeste homolog 2
EDTA: Ethylenediaminetetraacetic acid
OD: Optical density
RT-qPCR: Reverse transcription quantitative polymerase chain reaction
GAPDH: Glyceraldehyde-3-phosphate dehydrogenase
RIP: RNA immunoprecipitation
FISH: Fluorescence in situ hybridization
